# Medical Books in Buffalo

**Published:** 1846-06-01

**Authors:** 


*Medical Books in Buffalo.*—One of the most important of the duties of the medical journalist, is to keep his readers informed of the new additions being constantly made to the Bibliographical literature of the profession. We design to make this, hereafter, an especial object; but in so doing, we must ask the co-operation of medical publishers. As it is a matter concerning *their* interests not less than the interests of our readers, we may with propriety suggest to them the policy of always sending copies for sale at a point which is a depot for the profession, not only in this immediate neighborhood, but, to a considerable extent, in the Western States, and Western Canada. We experience difficulty in having early access to new works, not being able to find them at our book stores. Our booksellers intend to keep an assortment of late and approved works; but we cannot, of course, take the responsibility of advising them to send for those the merits of which we have not been able to examine. We trust that not alone for our particular convenience, but for the interests of the profession, and their own advantage, medical publishers will take pains that all their new publications are duly represented in this city. We shall endeavor to bestow upon them all an impartial criticism, recommending them, when, in our opinion, the interests of the profession and our readers will be subserved by their circulation, but not hesitating to express views of a different character whenever the same interests (which we are bound chiefly to consult) seem to us to require their utterance.

